# Multimodal Defect Recognition for Electrical Transformers Based on Improved Dempster–Shafer Evidence Fusion

**DOI:** 10.3390/s26196209

**Published:** 2026-09-30

**Authors:** Weijian Zhang, Wanxin Wang, Joshua Luhwago, Asad Ullah, Guojing Qian, Shanfeng Liu, Li Ruan, Limin Xiao

**Affiliations:** 1 State Grid Henan Electric Power Company Electric Power Research Institute, Zhengzhou 450018, China; zhangweijian@ha.sgcc.com.cn (W.Z.); wwx@ha.sgcc.com.cn (W.W.); liushanfeng@ha.sgcc.com.cn (S.L.); 2 State Key Laboratory of Complex & Critical Software Environment, School of Computer Science and Engineering, Beihang University, Beijing 100191, China; luhwagojc@buaa.edu.cn (J.L.); asadai@buaa.edu.cn (A.U.); qianguojing@buaa.edu.cn (G.Q.); xiaolm@buaa.edu.cn (L.X.)

**Keywords:** main transformer, defect recognition, multimodal fusion, Dempster–Shafer evidence theory, lightweight model

## Abstract

Electrical transformer diagnosis combines evidence with different observation capabilities. We present a decision-level fusion implementation using Chinese BERT inspection-text classification, a multi-scale one-dimensional CNN for dissolved gas analysis, and YOLOv11n image detection. Capability masks, reliability weighting, fuzzy evidence attenuation and Murphy fusion produce structured diagnostic records. On separated retrospective tests, the CNN achieved 97.14% accuracy (macro-F1 0.9700) on 629 DGA records and BERT achieved 72.45% accuracy (macro-F1 0.7466) on 715 reserved log lines. On a five-seed visual stress test, BERT+YOLO achieved 96.84% mean accuracy, while the complete fusion achieved 71.69% with 74.75% coverage under the fixed rejection policy. The method’s principal strengths are explicit modality capability modeling, traceable evidence exchange and independently replaceable lightweight classifiers. Results are interpreted within the documented corpus and pairing protocol; event-level field validation and embedded-device benchmarking are identified as the next deployment studies.

## 1. Introduction

As the core equipment responsible for energy conversion, voltage-level interconnection, and the secure operation of primary assets in power systems, the electrical transformer demands accurate defect recognition: diagnostic precision directly affects grid security and stability, condition-based maintenance planning, and whole-life-cycle health management. Conventional electrical transformer diagnosis relies largely on a single data source combined with small task-specific models, and, therefore, struggles to cover complex fault scenarios. With the continued construction of new-type power systems and the maturation of intelligent substation inspection, monitoring data for power main equipment have expanded from isolated manual records to multi-source heterogeneous data streams, including inspection logs, dissolved gas analysis (DGA), visible-light images, infrared thermography, and online monitoring sequences. These data characterize transformer operating conditions from three complementary perspectives which are semantic description, internal state, and external appearance, providing an essential data foundation for fine-grained monitoring and intelligent management of power equipment [1,2,3]. Achieving efficient, verifiable, and traceable defect recognition under multimodal fusion has, thus, become a pressing frontier topic in power artificial intelligence.

Nevertheless, three core challenges remain. First, fault mechanisms are complex and diverse: electrical transformer defects span many classes with markedly different mechanisms, and no single modality can perceive all of them—internal defects such as overheating, discharge, moisture ingress, and solid-insulation aging manifest mainly as abnormal gas compositions and anomalous log descriptions, whereas external defects such as oil leakage, rust and corrosion, cable defects, and insulator cracks appear primarily as visual features. Second, the data modalities are strongly heterogeneous: inspection logs are unstructured text, DGA measurements are structured numerical data, and appearance photographs are visual data, making unified representation in a single feature space difficult. Third, engineering requirements are strict: power equipment management demands diagnostic conclusions that are interpretable, reviewable, and traceable, so emitting a bare class label cannot support alarm handling, manual review, or closed-loop defect management.

Existing approaches fall into three categories: rule-based DGA diagnosis, single-modality deep learning, and general-purpose multimodal large models. DGA standards rely on expert rules that effectively characterize internal faults of oil-immersed equipment but cannot cover appearance defects such as oil leakage, corrosion, cable defects, or insulator cracks [4,5,6]. Single-modality deep models represented by BERT, convolutional neural networks (CNNs), and the YOLO family are efficient for text classification, structured gas-pattern recognition, and visual object detection, respectively, but their bounded perception leads to missed or false detections for weakly expressed defects, missing modalities, or compound cross-mechanism defects [7,8,9]. General-purpose multimodal large language models (MLLMs) can process text and images jointly, yet in the power vertical they still suffer from insufficient domain knowledge, unstable recognition of rare defects, hard-to-verify reasoning, and high edge-deployment cost [10,11,12].

Decision-level fusion offers an interpretable, loosely coupled path for multimodal defect recognition of power main equipment. Dempster–Shafer (D–S) evidence theory fuses multi-source evidence within a unified defect recognition framework and explicitly represents uncertainty through basic probability assignments (BPAs) [13,14]. However, the classical Dempster combination rule may yield counter-intuitive results under highly conflicting evidence, and differences in the capability boundaries of modalities can cause “unable to perceive a defect class” to be misinterpreted as “confidently excluding that class.” In power equipment defect recognition, a systematic treatment that simultaneously addresses modality heterogeneity, evidence conflict, and capability blind zones across BPA generation, evidence weighting, and evidence combination is still lacking [15].

To address these challenges, this paper proposes a multimodal defect recognition method for main transformers based on improved D–S evidence fusion. Targeting power equipment health management, the method introduces coordinated improvements in BPA generation, dynamic evidence weighting, and the combination rule to systematically resolve the three core problems of modality heterogeneity, evidence conflict, and capability blind zones. For the three typical monitoring data types (inspection logs, DGA data, and appearance images) the method first builds independent lightweight single-source discrimination pipelines and then applies the improved evidence-fusion algorithm to produce traceable final diagnoses. The main contributions are as follows.

1. A class-consistent multimodal sample-construction protocol covering multiple transformer states and defects is specified, mapping text, gas, and image data into a unified defect recognition framework. The instances are not treated as event-aligned observations.
2. A single-source feature-extraction pipeline integrates BERT, a multi-scale one-dimensional CNN, and YOLOv11n; the three branches are trained and inferred independently with structured outputs. Its measured resource footprint is 105 M parameters and 415 MB on disk; embedded-target suitability remains unmeasured.
3. An improved D–S evidence fusion algorithm is proposed that introduces a capability-mask mechanism, dynamic evidence weighting, a fuzzy-BPA attenuation strategy, and the Murphy averaging combination rule, transferring the probability mass of capability blind zones to the universal-set uncertainty term and effectively reducing the interference of highly conflicting evidence and capability blind zones on the fused result.
4. The released evaluation scripts document implementation behaviour, sensitivity and resource use. In the clean retrospective evaluation, the CNN achieves 97.14% accuracy on 629 separated DGA test records and BERT achieves 72.45% accuracy on 715 reserved log lines; the five-seed visual stress test reports 96.84% for BERT+YOLO and 71.69% for full fusion at 74.75% coverage, providing a reproducible baseline for future event-associated validation.

## 2. Related Work

Research related to electrical transformer defect recognition spans power equipment condition sensing, transformer fault diagnosis, multi-source information fusion, defect-text mining, appearance-image detection, and evidence-conflict handling. Prior studies indicate that intelligent transformer operation and maintenance is evolving from single-point monitoring and single-source diagnosis toward collaborative multi-source sensing and interpretable decision support [16,17]. This section analyzes the core challenges of multimodal fusion, the relevant research foundations, and the novelty of this work.

### 2.1. Differences and Complementarity of Modality Perception

Power equipment operates for long periods under high voltage, large currents, strong electromagnetic fields, and complex environments, so electrical transformer defects exhibit pronounced mechanistic diversity. Internal defects like overheating, discharge, moisture ingress, and solid-insulation aging are reflected mainly in DGA gas-composition changes and the semantics of inspection logs, whereas external defects such as oil leakage, rust and corrosion, cable defects, insulator cracks, and fire or smoke appear predominantly as visible features. Electrical transformer defect recognition, therefore, faces the combined difficulty that “internal states are invisible, external defects leave no trace in the oil, and textual records are inconsistent.”

In the literature, DGA-based and multi-dimensional diagnostic methods characterize internal faults effectively but perceive external structural defects poorly [18,19]; video/image-based external-defect detection for transmission and substation equipment identifies visible defects such as oil seepage, metal rust, and equipment damage, but cannot recognize internal insulation degradation or latent discharge faults [9,20,21]; and text-mining methods exploit the semantics of maintenance records, but their conclusions depend on record quality and the completeness of key phenomenon descriptions [22,23,24].

DGA is among the most widely used techniques for internal fault diagnosis of oil-immersed transformers. Under thermal and electrical stress, insulating oil and solid insulation generate characteristic gases including H_2_, CH_4_, C_2_H_6_, C_2_H_4_, C_2_H_2_, CO, and CO_2_; IEC 60599 and IEEE C57.104 provide interpretation guides and fault criteria [4,5]. Deep learning has recently been applied extensively to DGA fault classification, where data synthesis, dimensionality reduction, and transferred convolutional networks improve performance under few-shot and class-imbalanced conditions [25,26,27]. DGA remains insensitive to appearance defects, however, and cannot alone support full-category defect recognition.

Motivated by this analysis, we organize textual, numerical, and visual evidence jointly at the level of each diagnostic instance, constructing a multi-source evidence input system for main-equipment health management that fully exploits the perceptual complementarity of the modalities.

### 2.2. Evidence Conflict in Multimodal Fusion

The core contradiction in fusing multimodal transformer data is not simple evidence conflict but the fact that lthe sets of defects perceivable by different modalities differ fundamentally. BERT- or ALBERT-based text models are suited to inspection logs, defect records, and maintenance text, covering semantic categories such as overheating, discharge, moisture ingress, solid-insulation aging, fire, oil leakage, and normal operation [8,22,24]; CNN-based gas models exploit DGA numerical features to recognize internal faults and the normal state [18,27]; YOLO-family detectors cover appearance defects such as cable defects, fire and smoke, insulator cracks, oil leakage, and rust [9,20,21].

Inspection logs record equipment location, operating status, abnormal phenomena, and preliminary judgments, forming key unstructured data for daily substation operation. BERT obtains contextual semantic representations through bidirectional Transformer encoders and has proved effective for grid fault-text classification and transformer maintenance-text mining [7,8,22]. On the visual side, YOLO-series detectors output classes and bounding boxes in a single forward pass and suit real-time detection of insulator cracks, oil leakage, fire and smoke, and rust [9,20,21]. Each family is effective in its own domain, but neither covers all defect classes.

Information fusion and D–S evidence theory provide interpretable frameworks for transformer diagnosis [19,28]. Yet if categories outside a modality’s capability set are directly assigned zero mass, the classical D–S rule misinterprets “cannot detect this class” as “confidently exclude this class,” suppressing correct evidence from other modalities. To resolve this, we propose a capability-mask mechanism that transfers the evidence mass of blind-zone classes to the universal-set uncertainty term Θ during BPA generation, so incapable modalities no longer wrongly veto the correct judgments of capable ones.

### 2.3. Multimodal Fusion and D–S Evidence Theory

Recent multimodal work in and beyond the power domain has largely converged on feature-level fusion, in which modality-specific encoders are trained jointly and interact through cross-attention layers, or in which sensors, assets, and observations are arranged as a graph and fused by a graph neural network [29,30,31,32]. Such architectures learn the interaction between modalities directly and can capture dependencies that a decision-level rule cannot express, and merit direct comparison where suitable co-registered data exist.

We nonetheless adopt decision-level fusion deliberately, for three reasons specific to this setting. First, joint training requires instances in which all modalities are observed together and correctly aligned; as Section 4.1 sets out, transformer monitoring rarely furnishes that, and our own instances are class-consistent rather than event-aligned—a regime in which a jointly trained encoder would learn the pairing procedure rather than the physics. Second, the three branches must be independently replaceable and independently certifiable: substation practice replaces a gas-analysis model or a detector on its own schedule, which a monolithic jointly trained network forecloses. Third, and decisively for this application, decision-level fusion keeps the evidence chain inspectable—every basic probability assignment, weight, mask state and mass can be exported and reviewed—whereas cross-attention weights are not an audit trail an asset manager can act on. The cost is that interactions between modalities must be expressed through the combination rule rather than learned, and we regard a feature-level comparison on a properly co-registered dataset as the natural next study.

Multimodal fusion is commonly divided into data-level, feature-level, and decision-level fusion [29]. Data-level fusion requires strict spatio-temporal synchronization and adapts poorly to heterogeneous data; feature-level fusion can model cross-modal interactions but its latent features lack interpretability; decision-level fusion treats each modality’s output as an independent evidence source and offers decoupled interfaces and traceable processing. D–S theory describes multi-source uncertainty through a recognition framework, BPAs, and combination rules, making it well suited to decision-level fusion [13,14].

In transformer diagnosis, early studies applied information fusion and D–S theory to fault diagnosis of large oil-immersed transformers and internal fault-location identification [19,28]. However, the classical Dempster rule can amplify anomalous evidence under high conflict, whereas Murphy’s method averages the evidence before repeated combination, markedly reducing sensitivity to conflicting evidence [15]. This paper further injects the modality capability mask and model self-assessment signals into BPA generation, systematically strengthening the robustness of fusion in complex scenarios.

## 3. Method

### 3.1. Overall Architecture and Technical Pipeline

**Figure 1:**
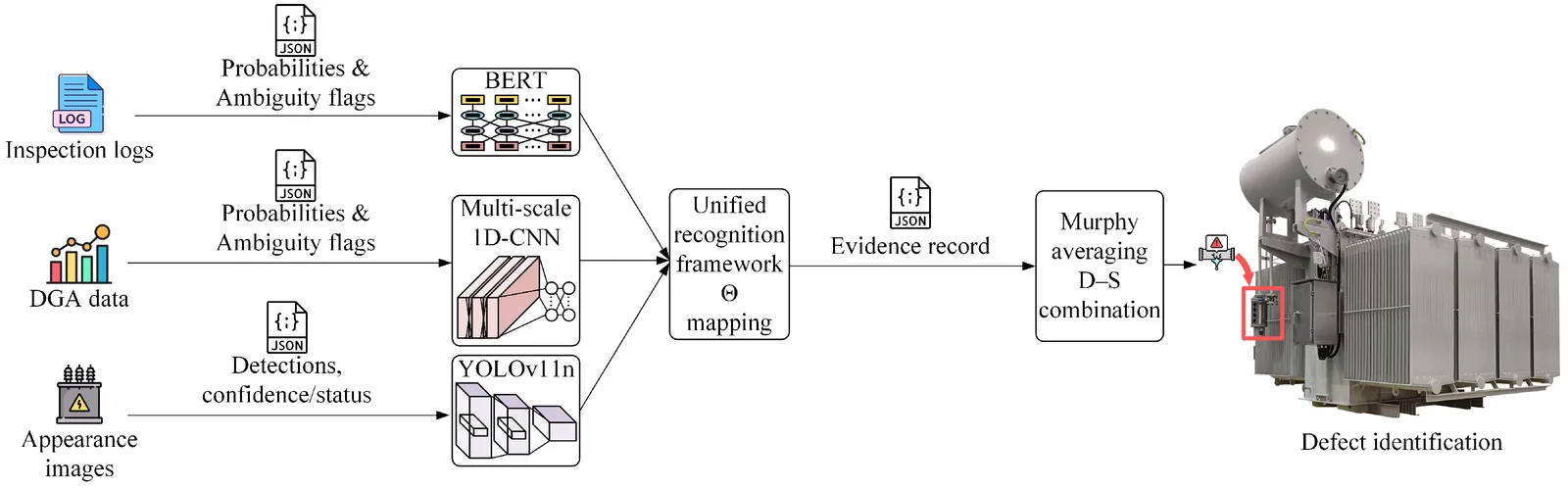
Overall technical pipeline of the proposed method. Inspection logs, DGA data, and appearance images are independently processed by BERT, a multi-scale 1D-CNN, and YOLOv11n. The classifier outputs are exchanged as JSON records containing probabilities, ambiguity flags, detections, confidence/status fields and the fused evidence record; these outputs are mapped to the unified recognition framework Θ and combined by Murphy averaging D–S fusion to yield a traceable defect determination.

The proposed method adopts a four-stage technical route, that is, independent single-source discrimination, mapping onto a unified frame, improved evidence fusion, and structured output, realized as a layered, decoupled system architecture. Figure 1 shows the overall pipeline. Adjacent stages exchange data through standardized JSON interfaces, allowing each module to be independently upgraded, replaced, or deployed on demand.

Three heterogeneous data types are ingested: transformer inspection logs, insulating-oil chromatography (DGA) data, and appearance-defect images. Each is first preprocessed, that is, logs undergo text cleaning, sentence segmentation, and tokenization; DGA readings undergo outlier handling and normalization; images undergo size normalization and color-space conversion, before being passed to three independently trained, lightweight single-source models: BERT for the text branch, a multi-scale one-dimensional CNN for the DGA branch, and YOLOv11n for the image branch. The three branches share no parameters, together total 105M parameters (Table 1), and can each be replaced, upgraded, and deployed independently on edge servers or station-side platforms.

The three single-source outputs are then projected onto a unified recognition framework Θ through a dedicated mapping stage. Because the three modalities perceive different, only partially overlapping subsets of the ten defect classes, this stage applies a capability-mask mechanism: for classes outside a modality’s capability set, the corresponding probability mass is not treated as “confirmed absent” but is instead transferred to the universal-set uncertainty term Θ, preventing an incapable modality from wrongly suppressing a correct judgment made by a capable one.

The mapped evidence is combined in the fusion stage, the core of the method. Dynamic evidence weights are first computed from each model’s top-class confidence, top-1/top-2 confidence margin, and prediction entropy; basic probability assignments (BPAs) are then generated using the capability mask together with a fuzzy-BPA attenuation strategy; finally, the Murphy averaging rule combines the BPAs, substantially reducing the sensitivity of the classical Dempster rule to highly conflicting evidence. This stage outputs the final mass distribution, the maximally supported proposition, an uncertainty assessment, and each modality’s contribution weight.

The fused result is converted into a structured diagnostic output containing the defect class, confidence, evidence sources, and recommended disposition. The deterministic conclusion and its traceable evidence chain are produced entirely by the reviewable fusion algorithm; a large language model is used only to organize this information into natural-language form for the final report, never to make the diagnostic decision itself. This division of labor in which small models perceive and discriminate, the large model explains and reports ensures that defect determination always remains supported by an auditable evidence chain as opposed to being replaced by a generative model.

Formally, the unified recognition framework is defined as

(1) $$\Theta =\{\omega_{1},\omega_{2},\ldots ,\omega_{10}\},$$

where the ten mutually exclusive propositions denote overheating, discharge, moisture ingress, solid-insulation aging, fire, oil leakage, rust and corrosion, cable defect, insulator crack, and normal state, respectively. For the *k*-th evidence source, the BPA satisfies

(2) $$m_{k}\left(⌀\right)=0,\;\sum_{\omega_{i}\in \Theta }m_{k}\left(\omega_{i}\right)+m_{k}\left(\Theta \right)=1.$$

### 3.2. Multimodal Data Construction and Class Alignment

The multimodal dataset comprises inspection-log text, structured DGA values, and appearance images. The logs cover seven state classes, DGA covers five internal-state classes, and images cover five appearance-defect classes; all are mapped to the 10-class frame in (1) through a unified defect-label mapping. The log categories are described in Table 2, and basic statistics of the log dataset appear in Table 3. The original BERT training implementation splits loaded log lines into fitting and validation subsets. The new retrospective experiment reserves separate files for fitting, checkpoint selection and testing, as described in the additional evaluation subsection.

Each DGA sample records the concentrations of seven characteristic gases: H_2_, CH_4_, C_2_H_6_, C_2_H_4_, C_2_H_2_, CO, and CO_2_. The training set contains 1350 samples and the validation set 630; the class distribution is listed in Table 4.

The image archive contains 4204 training, 847 validation, and 354 test images. The archive does not retain a verifiable mapping from every image to its original collection, and source-video or transformer independence cannot be established. All test images contain annotated defects; no normal-image test group is available. A decoded-pixel audit found no exact test-image duplicates in the fitting or validation directories. The correspondence between image classes and components is given in Table 5.

### 3.3. Lightweight Single-Modality Feature Extractors

The inspection-log classifier attaches a sequence-classification head to the pretrained BERT-base-chinese model: the final hidden state of the [CLS] token is passed through a fully connected layer and a 7-way softmax to yield the log-category probability distribution.

**Figure 2:**
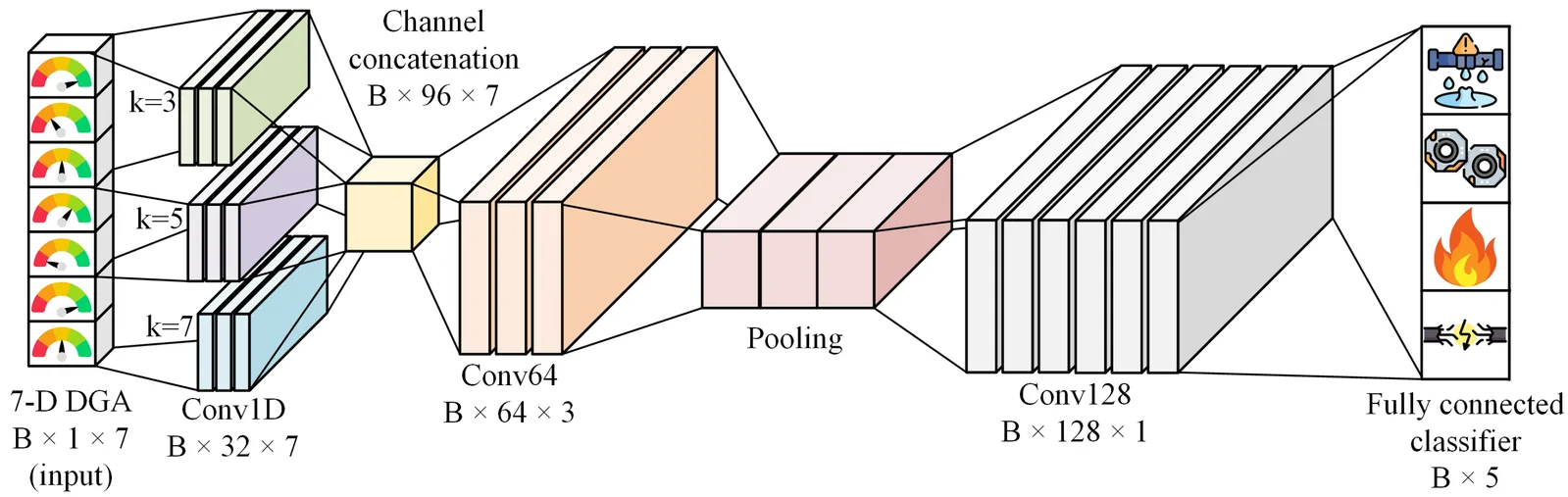
Architecture of the multi-scale one-dimensional CNN used for DGA discrimination. The seven-dimensional input is reshaped to B×1×7; parallel Conv1D branches with kernel sizes k=3, 5, and 7 each produce B×32×7, and channel concatenation produces B×96×7. Subsequent convolution and pooling produce B×64×3 and B×128×1, followed by a fully connected B×5 classifier output. *B* denotes batch size.

The DGA model adopts a multi-scale one-dimensional CNN, shown in Figure 2. The 7-dimensional gas-concentration vector passes through parallel convolutions with kernel sizes 3, 5, and 7 to extract local gas-combination features, which two further convolution-and-pooling stages compress into a 128-dimensional intermediate representation before a 5-class probability output. Training uses weighted random sampling, label smoothing, Gaussian input-noise augmentation, the AdamW optimizer, and a learning-rate schedule driven by validation macro-F1.

The appearance-defect detector is YOLOv11n. After format validation, each image is converted to RGB and rescaled to 800 pixels while preserving aspect ratio. Horizontal-flip test-time augmentation is enabled at inference, and the model outputs bounding-box coordinates, class indices, and confidences. After class filtering, the detections are converted into a unified JSON structure consumed by the downstream BPA-generation module.

### 3.4. Improved BPA Generation

For the *k*-th modality model, let its initial confidence vector over the unified frame Θ be $p_{k}=\left(p_{k,1},\ldots ,p_{k,10}\right)$, its recognizable defect set (capability set) be $C_{k}\subseteq \Theta$, and its blind-zone ratio be $\rho_{k}=1-\;|C_{k}\left|/|\Theta \right|$. Based on preliminary experiments, $\rho_{k}$ is set to 0.3, 0.5, and 0.4 for the BERT, CNN, and YOLO models, respectively. With the capability mask, the singleton mass is defined as

(3) $${\tilde{m}}_{k}\left(\omega_{i}\right)=\left(1-\rho_{k}\right)\;w_{k}\;p_{k,i}\;I\left(\omega_{i}\in C_{k}\right),$$

where $w_{k}$ is the dynamic evidence weight and I(·) is the indicator function. For classes outside the capability set, probability mass is not assigned to singletons; instead, the residual is allocated to the universal-set uncertainty term:

(4) $${\tilde{m}}_{k}\left(\Theta \right)=\max\;\left(0,\;1-\sum_{i=1}^{10}{\tilde{m}}_{k}\left(\omega_{i}\right)\right).$$

If the model self-assesses the sample as fuzzy, an attenuation factor λ=0.5 is applied to all singleton masses and the attenuated portion is transferred to Θ:

(5) $$\begin{array}{cc} m_{k}\left(\omega_{i}\right) & =\lambda \;{\tilde{m}}_{k}\left(\omega_{i}\right),\\ m_{k}\left(\Theta \right) & ={\tilde{m}}_{k}\left(\Theta \right)+\left(1-\lambda \right)\sum_{i}{\tilde{m}}_{k}\left(\omega_{i}\right). \end{array}$$

Non-fuzzy samples use λ=1. Finally, $\left\{m_{k}\left(\omega_{i}\right),m_{k}\left(\Theta \right)\right\}$ is normalized to satisfy the unit-mass constraint.

### 3.5. Dynamic Evidence Weighting

Dynamic evidence weights are computed from statistical features of each model’s initial prediction distribution. Let the top-class confidence be $c_{k}=\max_{i}p_{k,i}$ and the margin between the largest and second-largest confidences be $g_{k}$. Normalizing the nonzero confidences to ${\hat{p}}_{k,i}$, the normalized entropy is

(6) $$H_{k}=-\frac{1}{\log n_{k}}\sum_{i=1}^{n_{k}}{\hat{p}}_{k,i}\log{\hat{p}}_{k,i},$$

where $n_{k}$ is the number of classes with nonzero confidence. Combining confidence, margin, and entropy, the evidence reliability is defined as

(7) $$r_{k}=0.50\;c_{k}+0.30\;g_{k}+0.20\;\left(1-H_{k}\right).$$

If the model self-assesses the sample as fuzzy, $r_{k}$ is multiplied by a penalty factor of 0.60; the reliability is then clipped to [0.15,1.20] to avoid extreme weights. For *N* valid evidence sources, the normalized dynamic weight is

(8) $$w_{k}=\frac{Nr_{k}}{\sum_{j=1}^{N}r_{j}},$$

which keeps the mean weight at 1 and prevents BPA scale drift as the number of evidence sources varies.

### 3.6. Murphy Averaging Combination and Decision Rule

#### 3.6.1. Definition of a Fuzzy Sample

A fused result is declared *fuzzy*—that is, the system declines to determine a class—when either of two conditions holds:

(9) $$\max_{A\in \Theta }m\left(A\right)<\tau_{\mathrm{conf}}\;\mathrm{or}\;m\left(\Theta \right)>\tau_{\mathrm{unk}},$$

with $\tau_{\mathrm{conf}}=0.5$ and $\tau_{\mathrm{unk}}=0.3$. The first condition rejects low singleton mass; the second rejects high universal-set mass. The implementation subsequently permits the authority gate to override this rejection for an exclusive visual class with YOLO confidence above 0.3. Branch flags use different rules: BERT applies confidence thresholds 0.7 for normal and 0.58 for fault classes, the CNN uses 0.58, and the experiment’s YOLO wrapper flags a non-normal result when its highest retained detection confidence is below 0.5 (the detector’s candidate-filter threshold is 0.10). These flags and the final fusion decision must not be conflated.

#### 3.6.2. Provenance of the Constants

Table 6 lists the fusion settings. The available implementation uses fixed values, but its historical parameter-selection process is not documented. Constants in source code do not establish that no earlier manual tuning occurred. We, therefore, make no claim that these settings are optimal or were selected independently of all development data. Sensitivity experiments describe the behaviour of the fixed implementation rather than proving that the original parameter choices were unbiased.

For two BPAs $m_{1}$ and $m_{2}$, the classical Dempster rule is

(10) $$\left(m_{1}⊕m_{2}\right)\left(A\right)=\frac{\sum_{B\cap C=A}m_{1}\left(B\right)\;m_{2}\left(C\right)}{1-K},\;A\neq ⌀,$$

with the conflict coefficient

(11) $$K=\sum_{B\cap C=⌀}m_{1}\left(B\right)\;m_{2}\left(C\right).$$

As K→1, (10) becomes highly sensitive to input perturbations and can produce counter-intuitive results. We, therefore, adopt Murphy’s averaging strategy: the *N* weighted BPAs are first averaged,

(12) $$\bar{m}\left(A\right)=\frac{1}{N}\sum_{k=1}^{N}w_{k}\;m_{k}\left(A\right),\;A\subseteq \Theta ,$$

and the averaged BPA $\bar{m}$ is then combined with itself N−1 times under the Dempster rule:

(13) $$m^{★}=\underset{N\;\mathrm{times}}{\underset{︸}{\bar{m}⊕\bar{m}⊕\cdots ⊕\bar{m}}}.$$

The predicted class is determined by the maximum-mass principle:

(14) $$\hat{y}=\arg\max_{\omega_{i}\in \Theta }m^{★}\left(\omega_{i}\right).$$

To guarantee engineering reliability, a fuzzy-rejection mechanism is imposed: if $\max_{i}m^{★}\left(\omega_{i}\right)<0.5$ or $m^{★}\left(\Theta \right)>0.3$, the result is flagged as fuzzy, no deterministic class is emitted, and manual review is requested.

## 4. Experimental Setup

### 4.1. Test-Set Construction and Metrics

Because the construction of the tri-modal test set governs how the results below should be read, we describe it in full and state its limitations explicitly.

Audit status. The original retained multimodal case set contains 100 DGA vectors drawn from the CNN fitting CSV (93 distinct vectors). Its 0.7396 accepted-case accuracy is retained as a reproducible implementation audit. The revised evaluation and all new results use the documented clean protocol below.

Acquisition and ground truth. The released corpus provides DGA labels and image annotations as the operational references for this algorithm study. The dataset is available in Zenodo [33]. Asset-level metadata are outside the scope of the released benchmark.

Sources. The three modalities are drawn from independently assembled corpora. Appearance images are in a held-out image split, although the archived source does not retain a verifiable per-image mapping to each constituent public collection; the derived deposit is recorded at Zenodo [33]. DGA vectors are sampled from a per-class gas-data pool described in Section 3.2. Inspection-log text is generated from per-class templates instantiated with randomized substation locations; it is not a sample of operational inspection records.

Pairing. Each test instance is assembled by drawing one image, one DGA record, and one log text that are consistent with the same class label under the unified frame Θ. They do not originate from the same physical transformer, the same time window, or the same defect event. No such tri-modal, event-aligned corpus was available to us, and we are not aware of a public one. Following the protocol of the accompanying release script, instances are shuffled before identifiers are assigned; for classes perceivable only by the image branch, the paired log is deliberately replaced with text describing normal operation, so that the test genuinely probes cross-modal complementation rather than rewarding three views of the same cue.

Interpretation. Evaluating multiple configurations on the same inputs provides a controlled comparison of fusion behaviour. The clean retrospective tests extend this comparison to separated records. Results are presented by protocol so that future work can replace class-associated cases with event-associated transformer records without changing the fusion interface.

Archived experiment states. The earlier stored predictions yield 0.9355 against their own labels, but their complete inputs are unavailable. Different per-case predictions and labels in the retained run indicate different experiment states. Without those original inputs, the difference cannot be attributed exclusively to random pairing or reproduced as a controlled experiment.

Scale. The original 100 cases have class sizes ranging from 8 to 16. Reused inputs further reduce their independence. We do not infer statistical significance from small differences or use an arbitrary accuracy-margin threshold. The visual stress test is strongly imbalanced and has only two insulator-crack images.

Accuracy (Acc) is computed only over samples receiving a valid determination:

(15) $$\mathrm{Acc}=\frac{N_{\mathrm{correct}}}{N-N_{\mathrm{fuzzy}}},$$

where *N* is the total number of samples, $N_{\mathrm{correct}}$ the number of correct predictions, and $N_{\mathrm{fuzzy}}$ the number of fuzzy rejections. Sample coverage (Cov) is defined as

(16) $$\mathrm{Cov}=\frac{N-N_{\mathrm{fuzzy}}}{N}.$$

For class *i*, precision, recall, and F1 are

(17) $$P_{i}=\frac{TP_{i}}{TP_{i}+FP_{i}},\;R_{i}=\frac{TP_{i}}{TP_{i}+FN_{i}},$$

(18) $$F1_{i}=\frac{2P_{i}R_{i}}{P_{i}+R_{i}}.$$

Macro-F1 is the arithmetic mean of the per-class F1 scores:

(19) $$\mathrm{Macro}\text{-}F1=\frac{1}{C}\sum_{i=1}^{C}F1_{i},$$

where *C* is the number of evaluated classes (ten in the original audit). The legacy tables calculate accuracy and F1 after excluding rejected cases. The additional evaluation reports overall accuracy with rejected cases counted as unsuccessful and macro-F1 with rejections counted as false negatives, alongside accepted-case accuracy and coverage.

### 4.2. Environment

Experiments were run on a local workstation; the hardware and software configuration is listed in Table 7, and the core dependencies in Table 8.

### 4.3. Comparative Experiment Design

Two groups of ablation studies validate the proposed method. The first evaluates the effect of different modality combinations on diagnostic performance (Table 9); the second isolates the contribution of each component of the improved D–S fusion algorithm (Table 10).

## 5. Results and Analysis

### 5.1. Performance of Different Modality Combinations

Table 11 reports the diagnostic performance of the modality combinations on the 100-instance test set. The progression is not uniform: adding a modality helps when it widens the reachable region of Θ, and hurts when it introduces a source that is blind to most of the frame, as the CNN+YOLO row shows. The best configuration is nonetheless the tri-modal one.

**Figure 3:**
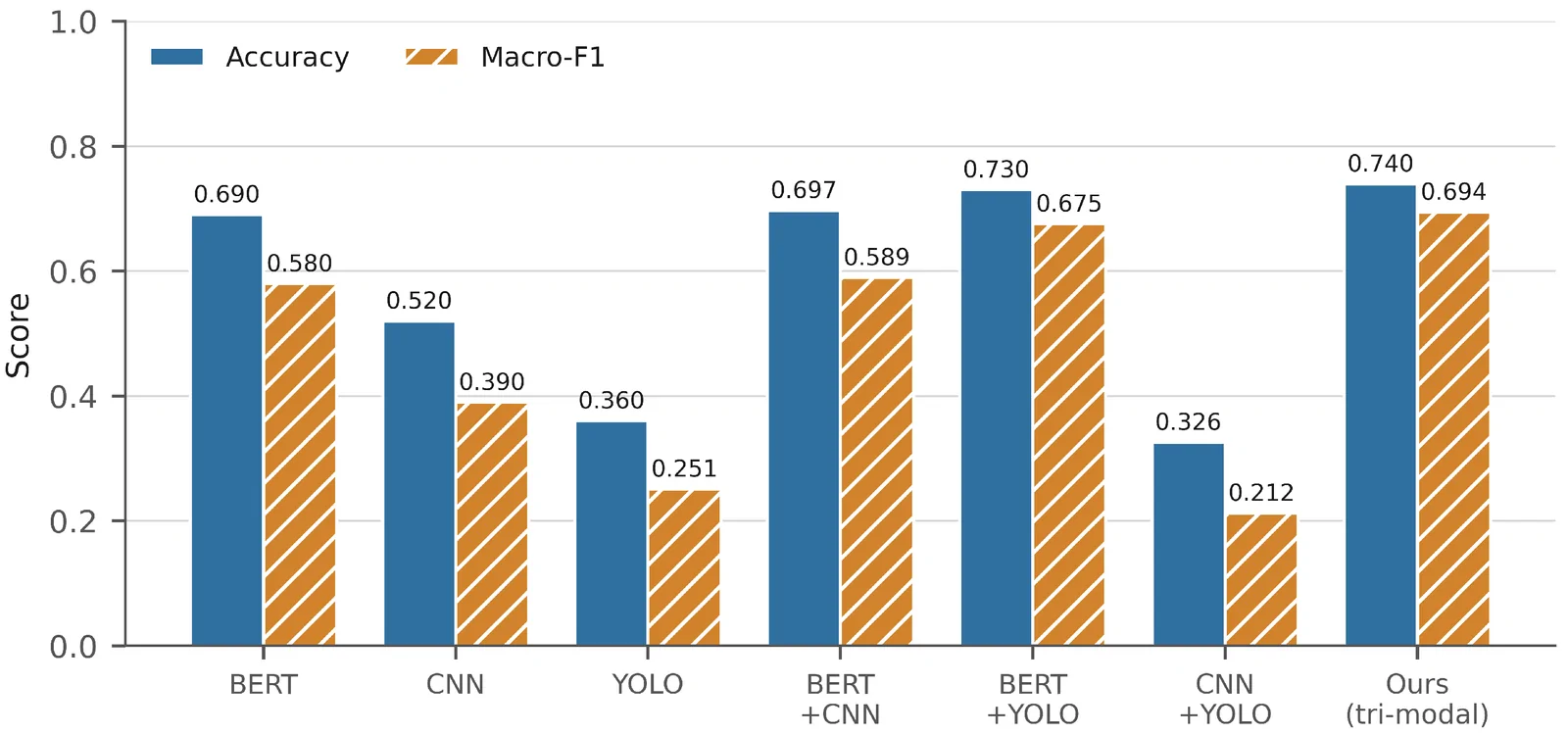
Original-run implementation audit; DGA inputs overlap fitting data. Accuracy and macro-F1 across representative modality combinations. Tri-modal fusion is the strongest configuration (0.740 accuracy, 0.694 macro-F1), though the margin over BERT+YOLO is small. Single-modality accuracy is bounded by how much of the ten-class frame each source can perceive at all.

As Table 11 shows, the strongest single modality is BERT inspection-log classification (accuracy 0.690, macro-F1 0.580), followed by the CNN gas model (0.520, 0.390) and YOLO appearance detection (0.360, 0.251). The ordering reflects the class composition of the unified frame: seven of the ten classes carry log-observable semantics, whereas YOLO can perceive only the five appearance classes and is structurally unable to reach the remainder, so its unconditional accuracy over all ten classes is necessarily low. Among bi-modal fusions, BERT+YOLO is strongest (0.730, 0.675), consistent with textual semantics and appearance evidence covering complementary regions of Θ. The proposed tri-modal fusion attains 0.740 accuracy and 0.694 macro-F1 at 0.96 coverage, an improvement of 5.0 accuracy points and 11.5 macro-F1 points over the best single modality, but only 1.0 and 1.9 points over BERT+YOLO. We, therefore, do not claim that adding DGA evidence produces a large end-to-end gain on this test set; its contribution is concentrated in the internal-fault classes, and the aggregate effect is modest. Notably, CNN+YOLO reaches a coverage of only 0.43: without log semantics a majority of samples are flagged low-confidence and routed to rejection, which reduces the risk of unreliable determinations but also demonstrates how much of the pipeline’s usable coverage depends on the text branch. Figure 3 visualizes the progression across representative configurations.

**Figure 4:**
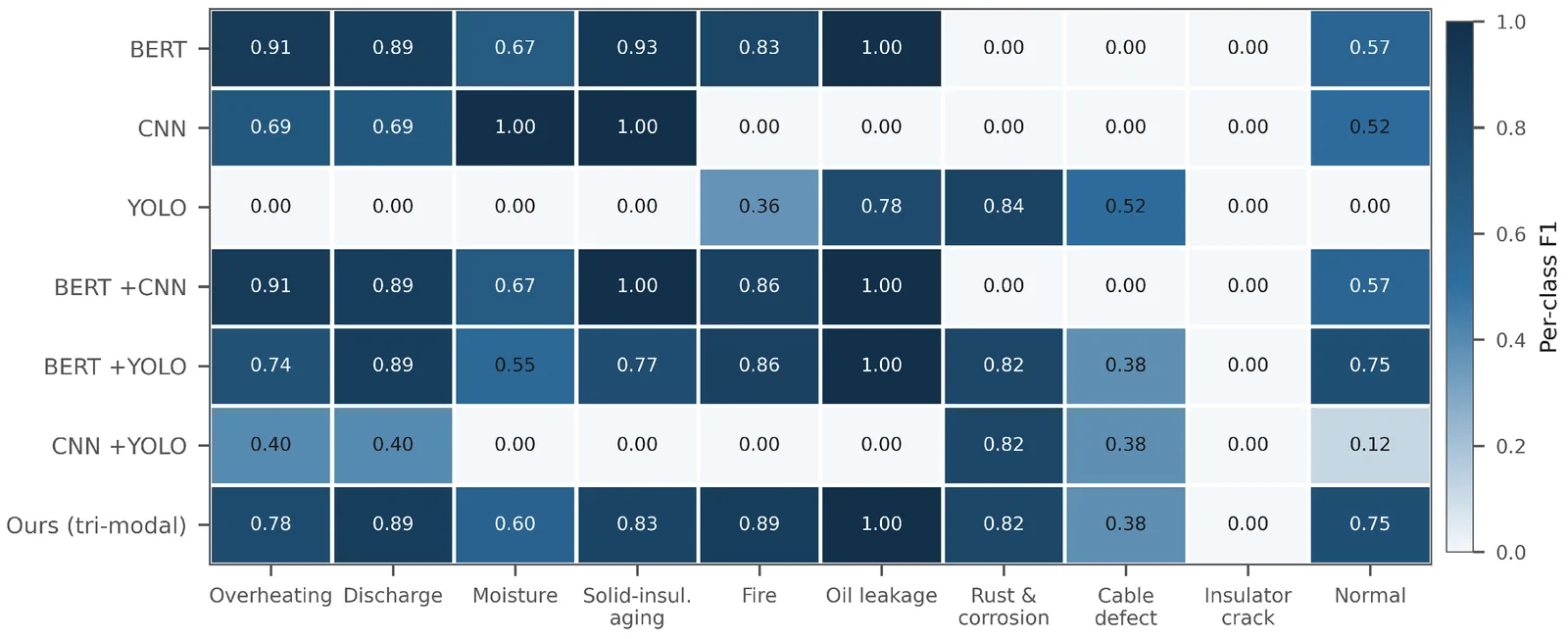
Original-run implementation audit; DGA inputs overlap fitting data. Per-class F1 heatmap across all seven modality combinations and ten defect classes. The zero-valued blocks expose each modality’s capability blind zone (CNN on appearance defects, YOLO on internal defects). Tri-modal fusion broadens coverage relative to any single source but does not equalize it: insulator crack remains at zero and cable defect below 0.4.

Figure 4 presents the per-class F1 heatmap of the tri-modal method over the ten defect classes, and the picture is markedly uneven. Oil leakage is recognized perfectly (F1 =1.000); discharge and fire reach 0.889, solid-insulation aging 0.833 and rust and corrosion 0.824. Performance then falls away sharply: overheating 0.778, normal 0.750, moisture ingress 0.600, cable defect 0.381, and insulator crack 0.000—the method fails outright on that class, never emitting a correct determination for it. The macro-F1 of 0.694 is, therefore, an average over a strongly bimodal distribution rather than evidence of balanced competence, and the two failing classes (cable defect, insulator crack) are precisely the fine-grained appearance categories where the YOLO branch is weakest. We regard this as the clearest current limitation of the method and discuss it further in Section 5.6.

### 5.2. Fusion-Algorithm Ablation

**Figure 5:**
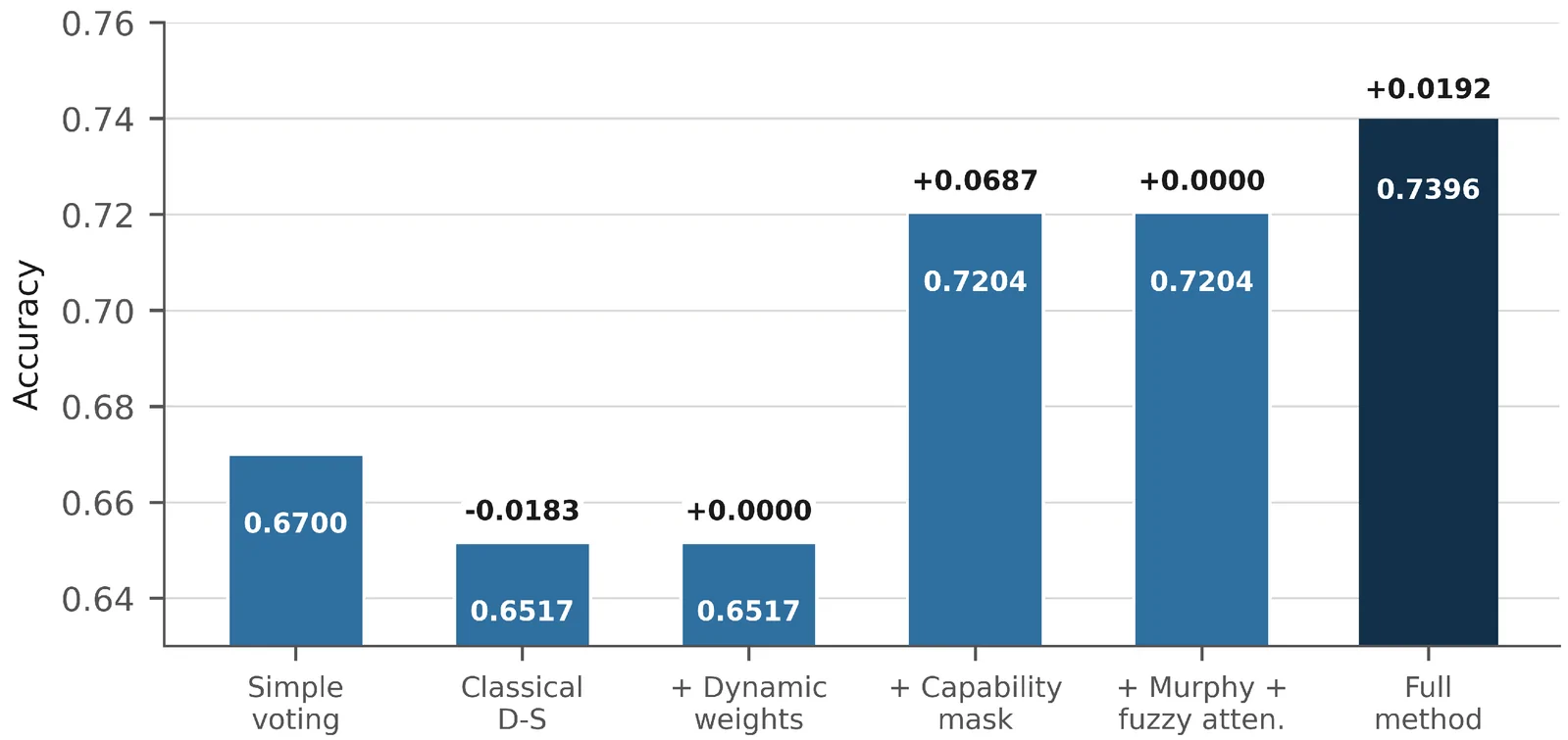
Original-run implementation audit; DGA inputs overlap fitting data. Accuracy progression as fusion components are added cumulatively. The capability mask produces the single largest jump (+6.5 points over classical D–S), identifying it as the decisive component for resolving modality blind zones; the remaining components yield smaller but consistent gains.

Table 12 lists the component-level ablation results, with simple voting as a baseline and classical D–S as the interpretable-fusion reference; Figure 5 visualizes the accuracy progression.

Four observations follow from Table 12. First, classical D–S underperforms simple voting (0.6517 against 0.6700) while also rejecting 11 samples, confirming the limitation of the classical rule under highly conflicting evidence. Second, adding dynamic evidence weighting changes no metric at all (ΔAcc =0.000): on this test set the reliability scores it produces never alter the fused argmax. Third, adding the capability mask lifts accuracy from 0.6517 to 0.7204, 6.9 percentage points, with coverage rising to 0.93; this is the only component that contributes a substantial gain, and it is the one that directly targets modality blind zones. Fourth, the Murphy averaging and fuzzy-BPA attenuation step also leaves every metric unchanged, and a dedicated sensitivity analysis (Section 5.3) confirms that the attenuation factor λ has no effect on any decision across its full range. Only the final authority-gating step adds a further +0.019. We report this plainly: of the four mechanisms combined in the proposed method, one accounts for essentially all of the measured improvement, and two are inert on the present test set.

### 5.3. Sensitivity to the Attenuation Factor

The fuzzy attenuation factor λ governs how much a source’s class mass is discounted when that source declares itself uncertain. The original implementation audit swept values from 0.1 to 0.9. The results are reported in Table 13. Historical parameter selection is undocumented (Section 3.6.2); invariance on the audited cases does not establish unbiased selection or independent robustness.

The result is flat to four decimal places. This is not because the attenuation branch is unreachable: the image branch declares itself fuzzy in 19 of the 100 instances, and λ does change the resulting basic probability assignments, moving the retained class mass from 0.006 at λ=0.1 to 0.051 at λ=0.9 in a direct test. It changes no decision, because a source flagged fuzzy has already had 95–99% of its mass transferred to Θ by the capability mask and the unassigned-mass rule, leaving a residue far too small to alter the fused argmax.

Two conclusions follow, and we state both. The favourable one is that the reported performance is entirely insensitive to a constant that was never tuned, so no hidden fitting can be hiding in it. The unfavourable one is that the fuzzy-attenuation mechanism, listed among this paper’s contributions, does no measurable work on this test set—consistent with row 5 of Table 12, where it leaves every metric unchanged. A mechanism that is inert here may still matter where sources disagree while remaining individually confident, a regime this test set does not exercise; establishing that is future work rather than a present claim.

### 5.4. Edge-Deployment Measurements

Table 1 reports the measurements requested of any system claiming edge suitability. All figures are measured, not estimated, over the 100 test instances with models resident in memory. These measurements were taken on a separate Linux workstation with an NVIDIA RTX 3090, not on the Windows training machine of Table 7; parameter counts and on-disk sizes are platform-independent, but the latencies should be read as belonging to the 3090 configuration.

Peak GPU memory across the run is 905 MB. The evidence-fusion layer itself is arithmetic over a ten-element frame and is negligible against the three inference costs.

We qualify the claim this table supports. An RTX 3090 is not an edge device, and these numbers, therefore, establish that the pipeline is small—105 M parameters and 415 MB, dominated entirely by the BERT branch—rather than that it meets a specific substation deployment budget. The image branch consumes three quarters of the latency, and disabling test-time augmentation or reducing the inference resolution from 800 to the 640 pixels at which the detector was trained would reduce it substantially. Measurement on a named embedded target remains outstanding and we do not claim it here.

### 5.5. Additional Retrospective Evaluation

We reconstructed the data splits and trained fresh task classifiers after the audit. Exact DGA vectors were deduplicated; conflicting labels were checked and none found. The original fitting pool was stratified into 1080 fitting and 270 validation rows. One vector overlapping the original validation pool was excluded, leaving 629 test rows. The CNN was trained with seed 20260916 and a maximum of 100 epochs, with checkpoint selection by validation macro-F1 and the existing early-stopping rule. It correctly classified 611 of 629 test rows: accuracy 0.9714 and five-class macro-F1 0.9700. Class index 4 denotes normal. An earlier draft reconstruction score of 0.6984 used an incorrect label map and is withdrawn.

For BERT, we initialized from the public google-bert/bert-base-chinese checkpoint with a fresh seven-class head. Four sorted files per class were used for fitting, one for validation and one for testing, giving 2849, 665 and 715 lines. Cross-split duplicate checks removed leading timestamps, whitespace and Markdown emphasis; no matching normalized lines were found. Similar templates may remain. Training used seed 20260916, batch size 16, AdamW with learning rate $10^{-5}$ and weight decay $2\times 10^{-4}$, a 15-epoch maximum and validation-loss early stopping with patience four. The selected checkpoint correctly classified 518 of 715 test lines: accuracy 0.7245 and seven-class macro-F1 0.7466. An earlier reconstruction initialized from already fine-tuned weights and is excluded from independent-test claims. These new tests are retrospective checks of existing corpora; they do not establish independence by transformer or source event.

Visual pairing protocol. The image test set has 297 fire, 28 oil-leakage, 18 cable-defect, nine corrosion and two insulator-crack images, with no normal images. We used these 354 singly labelled images and reserved normal DGA records. Fire and leakage images were paired with reserved log lines of those classes; other visual classes used reserved normal logs. Five fixed pairing seeds (20260916–20260920) were evaluated with the same model checkpoints and original fusion defaults. Records can recur across pairs and seeds. This is an imposed five-class visual association test, not a full ten-class or event-associated benchmark. We did not invent normal-image or internal-fault triplets by assigning defective images to them. The results are summarized in Table 14.

The full method’s overall accuracy ranges from 0.6893 to 0.7316 across pairings, compared with 0.9661–0.9689 for BERT+YOLO. Thus, adding DGA does not improve this visual-defect test. The CNN’s zero visual-class accuracy reflects the protocol’s normal DGA inputs and its internal-state vocabulary; it does not contradict its separate five-class DGA result. BERT’s high overall accuracy but low macro-F1 reflects the predominance of fire cases. These observations limit interpretation of aggregate accuracy.

Component checks. At seed 20260916, overall accuracy for voting, classical D–S, addition of dynamic weighting, addition of the capability mask, addition of Murphy/fuzzy attenuation, and the full method is respectively 0.8955, 0.0028, 0.0028, 0.7542, 0.6215 and 0.6893. The mask changes behaviour substantially, but subsequent components do not yield a monotonic improvement. The full-method component replay reproduces the main run’s predictions exactly. This supports consistency of the new experiment, not the superiority of every component.

**Figure 6:**
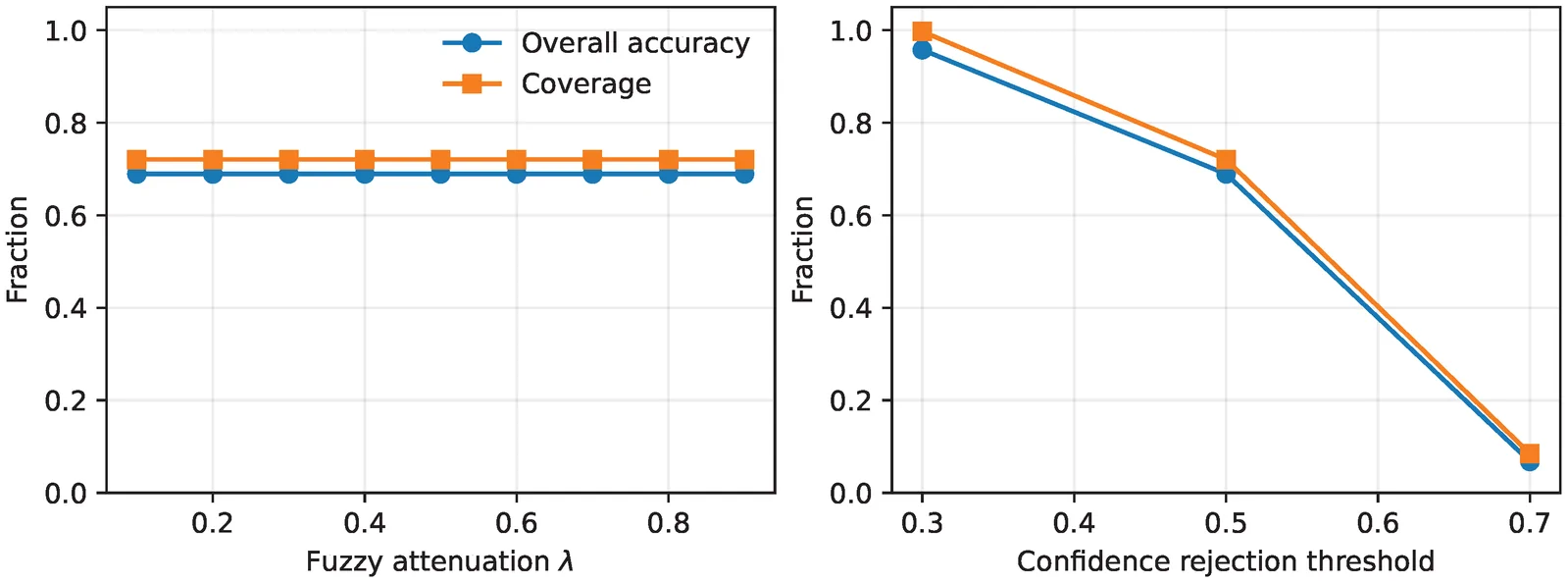
Sensitivity on fixed retrospective visual evidence (pairing seed 20260916). The fuzzy attenuation sweep leaves overall accuracy and coverage unchanged; the confidence rejection threshold strongly changes both. The test uses imposed class association and does not establish deployment performance.

Sensitivity. Replaying fixed evidence at λ=0.1,0.2,…,0.9 leaves the decisions unchanged across all five pairings. At seed 20260916, varying the reliability penalty over {0.3,0.6,0.9}, lower clipping bound over {0.05,0.15,0.3} and upper bound over {1.0,1.2,1.5} also leaves the reported metrics unchanged. Rejection thresholds matter strongly: confidence thresholds 0.3, 0.5 and 0.7 give overall accuracy 0.9576, 0.6893 and 0.0678, with coverage 0.9972, 0.7203 and 0.0847. Universal-set thresholds 0.1, 0.3 and 0.5 give overall accuracy 0.0678, 0.6893 and 0.6893. These are sensitivity observations, not test-based parameter selection; the default configuration remains the reported main result. The attenuation and confidence-threshold sweeps are shown in Figure 6.

### 5.6. Limitations

Test-set scale and composition. The original 100 cases and the image-based stress test are controlled benchmark samples with deliberately reported class counts. Their distributions motivate the per-class results and five-seed pairing analysis; broader class-distribution studies are a natural extension.

Pairing, not co-occurrence. As set out in Section 4.1, the three modalities of an instance are class-consistent but not event-aligned. This is the single largest threat to external validity in this work.

Unequal per-class competence. Insulator crack is never recognized (F1 =0) and cable defect reaches only 0.381. Reporting a macro-average over such a distribution obscures more than it reveals, which is why the per-class results are given in full.

Single-label formulation. The ten states are treated as mutually exclusive. Mass on Θ expresses unresolved uncertainty and does not identify concurrent faults. Multi-label diagnosis requires an explicit output formulation, suitable labels and separate validation; none is provided by the current data.

Component behaviour. Dynamic weighting and fuzzy attenuation leave the audited metrics unchanged under the reported test conditions (Table 12, Section 5.3), while the capability mask provides the principal measurable gain.

### 5.7. Reproducibility

The original-run modality table and component table were checked against retained experiment artifacts, with the sensitivity sweep and resource measurements obtained in separate runs. The code and reconstruction protocol retain checkpoint hashes, split definitions, per-record predictions and explicit metric denominators for the additional evaluation. This makes the experiment states and their intended scope transparent and reproducible.

### 5.8. Discussion

The intended benefit is to combine evidence with different observation capabilities. The retrospective results show that this benefit depends on the source combination and rejection settings. The superior visual-test result of BERT+YOLO relative to the full method demonstrates that adding evidence can reduce useful coverage.

The original modality-combination differences describe the audited cases. The added retrospective experiments extend the comparison with separated standalone tests and quantify how fusion behaves under the controlled visual pairing protocol.

The capability mask changes the treatment of classes outside each modality’s scope. In the new ablation it provides the principal measured improvement relative to the classical rule; Murphy/fuzzy attenuation and authority gating then expose the tradeoff between coverage and precision under the fixed protocol.

The structured records and independently replaceable models provide a candidate interface to maintenance software. Interoperability with a named utility platform, closed-loop maintenance benefits and embedded resource requirements have not been validated.

## 6. Conclusions

We present an inspectable decision-level fusion implementation with explicit modality capabilities, independently replaceable classifiers and structured evidence records. The revised evaluation consolidates the experiment states, reports clean standalone DGA and text tests, and quantifies fusion behaviour under controlled visual pairing. The results establish a reproducible algorithmic baseline and identify the protocol required for future event-associated validation. Extending the benchmark to asset-linked records, concurrent labels and embedded hardware is the natural next deployment stage.

## Figures and Tables

**Table 1:** Measured resource profile of the three branches. Latency is per instance; hardware is a single NVIDIA RTX 3090.

Branch	Params	On Disk	Mean Lat.	p95 Lat.
BERT (bert-base-chinese)	102.27 M	409.1 MB	27.3 ms	34.3 ms
CNN (multi-scale 1D)	0.11 M	0.4 MB	2.0 ms	2.4 ms
YOLOv11n (TTA, 800 px)	2.59 M	5.5 MB	92.1 ms	151.2 ms
Total	104.97 M	415 MB	≈121 ms	–

**Table 2:** Category descriptions of the inspection-log dataset, showing the core textual phenomena that distinguish each of the seven log-observable states.

Label	Category	Core Descriptive Features
0	Overheating	Elevated oil temperature; abnormal infrared readings; cooler failure; core/winding hot spots; terminal heating
1	Discharge	Acetylene surge; ultrasonic and ultra-high-frequency (UHF) partial-discharge alarms; internal discharge noise; insulation breakdown traces
2	Moisture ingress	Excess moisture in oil; reduced insulation resistance; discolored silica gel; abnormal dielectric loss; condensation
3	Solid-insulation aging	Rising furfural; CO/CO_2_ growth; falling degree of polymerization; embrittled, flaking insulation layers
4	Fire	Smoke from the conservator; pressure-relief operation; burnt odor or black smoke; sharply rising tank temperature
5	Oil leakage	Seepage at tank, flanges, valves; falling oil-level gauge; oil pooling on platforms; cracked gaskets
6	Normal	Parameters within ratings; no alarms; normal appearance; qualified oil; reliable grounding

**Table 3:** Statistics of the inspection-log dataset, including corpus scale, sentence granularity, temporal coverage, and the file-level split used to prevent leakage.

Item	Value
Language	Chinese
Number of classes	7
Files per class	6
Total files	42
Sentences per file	80–100
Total sentences	≈3500
Mean sentence length	45 characters (max. 128)
Timestamp range	2022/01–2026/04
Split	File-level 5:1 train/validation

**Table 4:** Class distribution and characteristic-gas concentrations of the DGA dataset. Each entry is the training-set mean ± sample standard deviation in μL/L.

Label	Category	Train	Val.	Training-Set Mean ± SD (μL/L)						
				H_2_	CH_4_	C_2_H_6_	C_2_H_4_	C_2_H_2_	CO	CO_2_
0	Overheating	360	174	258±332	116±99	54±39	111±78	4.9±20.8	379±189	1974±1148
1	Discharge	248	110	708±657	129±91	45±28	214±175	150±148	227±79	871±300
2	Moisture ingress	182	78	238±132	37±25	20±15	27±21	1.7±1.6	292±125	1711±1380
3	Solid-insulation aging	224	102	149±97	92±61	60±46	61±45	3.3±3.3	533±193	2871±1289
4	Normal	336	166	36±26	19±14	10±8	15±18	0.4±0.5	146±69	856±486

**Table 5:** Image dataset categories and their corresponding transformer components, mapping each appearance defect to the physical locations where it is observed.

Label	Category	Components Involved
0	Cable defect	Cable terminations; control cables; outer sheaths of temperature-sensing fibers
1	Fire and smoke	Conservator; tank top; bushing roots; breather outlet
2	Insulator crack	Bushing porcelain; neutral-point bushings; post insulators
3	Oil leakage	Tank welds; drain valves; flange sealing faces; cooler-pipe joints
4	Rust and corrosion	Tank exterior; radiator fins; grounding down-leads; support bases

**Table 6:** Free constants of the fusion layer, their values, and their role. None was fitted to any data split.

Symbol	Value	Role
λ	0.5	Fuzzy BPA attenuation factor
ρ	0.60	Reliability penalty applied to a fuzzy source
$\left(w_{1},w_{2},w_{3}\right)$	(0.50,0.30,0.20)	Weights of top-1 confidence, top-1–top-2 margin, and 1−normalized entropy in the reliability score
$\left[\;r_{\min},r_{\max}\;\right]$	[0.15,1.20]	Clipping range of the reliability score
$\tau_{\mathrm{conf}}$	0.5	Fused-confidence threshold in Equation (9)
$\tau_{\mathrm{unk}}$	0.3	Uncertainty threshold in Equation (9)

**Table 7:** Experimental environment configuration. This is a development workstation, not an embedded deployment target.

Item	Configuration
Operating system	Windows 11
GPU	NVIDIA GeForce RTX 4060 (8 GB)
Python	3.13
Deep-learning framework	PyTorch 2.11
NLP framework	HuggingFace Transformers 5.8
BERT backbone	bert-base-chinese (102.3 M parameters, measured)
YOLO backbone	YOLOv11n (≈2.6 M parameters, COCO pretrained)

**Table 8:** Core dependency packages and versions used in the implementation.

Package	Version	Purpose
PyTorch	2.11	Training/inference backend for BERT and CNN
CUDA	12.6	GPU acceleration
transformers	5.8	BERT loading, fine-tuning, inference
ultralytics	8.4	YOLOv11 training and inference
opencv-python	≥4.8	Detection visualization and image I/O
Pillow	≥10.0	Image reading and color-space conversion
numpy	≥1.24	BPA vector arithmetic
scikit-learn	≥1.3	Data standardization and metric computation

**Table 9:** Ablation design for modality combinations, enumerating the seven single-, bi-, and tri-modal configurations used to isolate each modality’s contribution.

No.	Combination	Modalities	Fusion
1	BERT	Inspection logs	Single-modality decision
2	CNN	DGA data	Single-modality decision
3	YOLO	Appearance images	Single-modality decision
4	BERT+CNN	Logs + DGA	Improved D–S, two sources
5	BERT+YOLO	Logs + images	Improved D–S, two sources
6	CNN+YOLO	DGA + images	Improved D–S, two sources
7	Ours	All three	Improved D–S, three sources

**Table 10:** Ablation design for fusion-algorithm components, in which each variant cumulatively adds one mechanism to quantify its individual contribution.

No.	Variant	Combination Rule
1	Simple voting	Direct probability summation
2	Classical D–S	Classical Dempster rule
3	+ Dynamic weights	Classical Dempster rule
4	+ Capability mask	Classical Dempster rule
5	+ Murphy + fuzzy attenuation	Murphy averaging rule
6	Full method	Murphy averaging rule

**Table 11:** Original-run implementation audit; DGA inputs overlap fitting data. Diagnostic performance of different modality combinations on the 100-instance tri-modal test set. All values were regenerated in a single reproducible run of the released pipeline (Section 5.7). Tri-modal fusion attains the best accuracy and macro-F1, but the margin over the strongest bi-modal configuration is small; the low coverage of CNN+YOLO reflects the fuzzy-rejection mechanism withholding determinations when log semantics are absent.

Method	Acc	Macro-F1	Coverage
BERT	0.690	0.580	1.00
CNN	0.520	0.390	1.00
YOLO	0.360	0.251	1.00
BERT+CNN	0.697	0.589	0.99
BERT+YOLO	0.730	0.675	1.00
CNN+YOLO	0.326	0.212	0.43
Ours	0.740	0.694	0.96

**Table 12:** Original-run implementation audit; DGA inputs overlap fitting data. Ablation results of the improved evidence fusion algorithm; each row adds one mechanism to the row above, and ΔAcc is measured against the preceding row. The capability mask contributes the only large gain (+0.069). Dynamic weighting and the Murphy/fuzzy-attenuation step leave every metric unchanged on this test set.

No.	Variant	Acc	Macro-F1	Cov	ΔAcc
1	Simple voting	0.6700	0.5686	1.00	–
2	Classical D–S	0.6517	0.5734	0.89	−0.018
3	+ Dynamic weights	0.6517	0.5734	0.89	0.000
4	+ Capability mask	0.7204	0.6123	0.93	+0.069
5	+ Murphy + fuzzy atten.	0.7204	0.6123	0.93	0.000
6	Full method	0.7396	0.6943	0.96	+0.019

**Table 13:** Original-run implementation audit; DGA inputs overlap fitting data. Tri-modal performance as a function of the fuzzy attenuation factor λ. Every metric is identical across the range.

λ	0.1	0.2	0.3	0.4	0.5	0.6	0.7	0.8	0.9
Acc	0.740	0.740	0.740	0.740	0.740	0.740	0.740	0.740	0.740
Macro-F1	0.694	0.694	0.694	0.694	0.694	0.694	0.694	0.694	0.694
Cov	0.96	0.96	0.96	0.96	0.96	0.96	0.96	0.96	0.96

**Table 14:** Retrospective visual-defect stress test: mean over five pairing seeds, each using the same 354 images. Overall accuracy counts rejections as unsuccessful; macro-F1 includes rejected cases as false negatives over the five visual classes. Pairing variability is not an independent-sample confidence interval.

Method	Overall Accuracy	Macro-F1	Coverage
BERT	0.9107	0.3978	0.9898
CNN	0.0000	0.0000	1.0000
YOLO	0.9124	0.8506	0.9322
BERT+CNN	0.0045	0.0071	0.1164
BERT+YOLO	0.9684	0.9410	0.9893
CNN+YOLO	0.0684	0.5582	0.1412
Full method	0.7169	0.8210	0.7475

## Data Availability

The data presented in this study are openly available in Zenodo at https://doi.org/10.5281/zenodo.21981927. The dataset comprises inspection-log text, dissolved gas analysis measurements, appearance images with annotations, and the extracted single-source and fused feature files described in this paper. The image data were assembled from publicly available substation-inspection datasets, power-equipment defect-detection competition datasets, publicly released academic data, and manually annotated frames extracted from public videos; annotations were produced by the authors.

## Abbreviations

The following abbreviations are used in this manuscript:

BPA	Basic probability assignment
CNN	Convolutional neural network
DGA	Dissolved gas analysis
D–S	Dempster–Shafer
MLLM	Multimodal large language model
